# Housing Spaces in Nine European Countries: A Comparison of Dimensional Requirements

**DOI:** 10.3390/ijerph18084278

**Published:** 2021-04-17

**Authors:** Letizia Appolloni, Daniela D’Alessandro

**Affiliations:** Department of Civil, Building and Environmental Engineering, Sapienza University of Rome, 00184 Rome, Italy; daniela.dalessandro@uniroma1.it

**Keywords:** housing, regulation, living spaces, housing dimensional requirements

## Abstract

Modern housing units must meet new needs and requirements; housing dimensions and functional characteristics are relevant issues, mainly considering population ageing and disability. The housing standards of nine European countries were compared to analyze their ability to satisfy new population need, in terms of size. The regulations were downloaded from the websites of the official channels of each country. A wide variability in room size was observed (e.g., single room: from 9 m^2^ in Italy to 7 m^2^ in France, to the absence of any limit in England and Wales, Germany-Hesse, and Denmark). Italian and French legislations define housing dimension considering the room destination and the number of people. The Swedish regulation provides performance requirements and functional indications but does not specify the minimum dimensions of habitable rooms. The rooms’ minimum heights vary between 2.70 m in Italy and Portugal and 2.60 m in the Netherlands, but no limits are established in England and Wales. A diverse approach among European countries regulations is observed: from a market-oriented logic one (e.g., England and Wales) in which room minimum dimensions are not defined to a prescriptive one (Italy) and one that is functionality-oriented (the Netherlands). However, considering the health, social, environmental, and economic trends, many of these standards should be revised.

## 1. Introduction

Today, modern housing units must meet new needs and requirements. These depend on various factors, such as the increase in average life expectancy of the population and the increase in people with related functional limitations, new social needs (foreign population, increase in separations and divorces, etc.) [1], new ways of working (e.g., remote working) and related technological needs, and the development of energy system requirements in response to climate change [2,3,4].

According to the World Health Organization (WHO), the home, in terms of living space, must be large enough to comfortably accommodate people of different ages, must guarantee sufficient space to fulfill the privacy needs of the occupants, and must be accessible and usable for external users [5]. The availability of sufficient space in the home, which guarantees the these requirements, is a fundamental aspect in the evaluation of housing quality [6].

This not only meets a basic human need for shelter, but also contributes to the physical and psychological well-being of the residents [6]. Although several authors [6,7,8,9] have dealt with various aspects of the quality of affordable housing in different countries, the discussion on housing standards and space planning is still rather open and leaves room for some considerations [10,11,12].

The availability of space in a home affects how and where people prepare and consume food, how they socialise, how they handle household waste and recycling, how they store goods, how much privacy they have for study, work, relaxation or free time, children’s play, to what extent and how they can adapt to new needs (e.g., isolation, disability), etc. [13,14].

A study carried out by the British Commission for Architecture and the Built Environment (CABE) in 2009 [15] found that 94% of the respondents believed that space in the home was a determining factor in their choice of where to live. Many of the respondents said they do not have enough space to carry out basic activities of their daily lives (spaces for preparing and preserving food, for socialising and entertaining guests, space for study, etc.).

Some authors [16] support the correlation between productivity and the opportunity to work from home. Therefore, an adequate space should meet environmental requirements, mainly related to the needs of person–space relationship based on the activities that are carried out within, but it should also meet psychological needs, hygiene requirements, safety, and full housing usability.

The impacts on health of inadequate living space are varied: several are associated with overcrowding, others with accessibility [2]. Many studies have reported a direct association between crowding and some negative health outcomes [17,18]. These mainly include infectious diseases and mental health problems [19,20]. Such problems lead to a clear inequality in health, as they occur mainly in the low-income population [21,22]. Overcrowding facilitates the spread of airborne infectious diseases (tuberculosis, influenza and related respiratory infectious diseases, meningococcal disease, Covid-19, etc.) and the spread of faecal-orally transmitted diseases [23,24]. The importance of increasing the distance [25] between people normally recommended in hospital environments has been, and is currently, the main preventive measure, both indoors (homes, offices, shops, etc.) and outdoors, to counteract the spread of Covid-19. In cases of overcrowding, however, it is not only the air transport that plays a role in the spread of the various infections, but also the direct personal contact and the indirect transmission through infected surfaces [26].

Interpersonal distance and spatial relationships between people and the environment play a fundamental role both in physical health (e.g., transmissible diseases) [17,19,20] and in feeling comfortable or uncomfortable in each situation. A review conducted by WHO [17] also found, via many longitudinal and cross-sectional studies, a significant association between household crowding and mental health problems. These mental health problems include psychological distress, alcohol abuse, depression and unhappiness, social isolation [17,27,28].

Reynolds’ 2005 study [27] carried out on 505 English families showed that more than 70% of those questioned considered overcrowding to be a factor affecting the health of family members; three-quarters of families living in severe overcrowding were also convinced that their children’s health has been affected by living conditions. For 86% of respondents, depression, anxiety, or stress were the result of poor living conditions.

Insufficient space does not provide a suitable environment for family life [29] and can strongly affect family relationships. Space shapes social interaction, determining how much interaction occurs and its results, positive or negative [29].

Critical issues related above all to the size and layout of housing have emerged strongly even during the lockdown due to the Covid-19 pandemic [3]; the overlapping of different activities (work, study, socialising, playing) in insufficient space can have a heavy impact on family life, creating a difficult dynamic in the relations between inhabitants of the same household [3,27].

Some studies [27,30] attributed a key role to space in determining the quality of family relationships, mainly due to lack of privacy. An adequate space contributes to personal privacy, reducing depression, anxiety, and stress, giving children space to play, and ensuring a good quality of sleep [31]. Children are more likely to be affected by overcrowding than the adult population, especially if they belong to low-income [10] families. In the UK, it has been estimated that overcrowding costs the British National Health Service (NHS) over £600 million per year [30].

Moreover, inadequate housing conditions can be related to symptoms of stress, anxiety, irritability, depression, even social misconduct (violence, vandalism, self-harm) [31] and alteration of attention capacities at school in children [32,33].

Cassen and Kingdon (2007) [34] highlight how the home learning environment can play a significant role in performance. Children’s activity is hampered (reduced school performance) by inadequate study space within the home, especially in areas where housing is of inferior quality. It is also accepted that stressful housing conditions can aggravate pre-existing psychiatric pathologies [32,35].

Regarding the health impacts associated with accessibility, failure to meet the occupants’ needs concerning the physical characteristics of the living space can generate impediments to the full use of the premises. In some cases, this can even represent a real source of danger since it can cause domestic accidents.

Housing dimensions and functional characteristics are relevant, mainly considering population ageing and disability. In the European Region, the number of people aged 85 years and older is projected to rise from 14 million in 2010 to 19 million by 2020 and to 40 million by 2050 [36].

As age increases, the percentage of people with disabilities also increases, making the accessibility and usability of homes crucial requirements to improve the ability of these people to live independently, using all indoor and outdoor spaces. This is an important issue, considering that environmental barriers (e.g., stairs and doorsteps) are present in most European homes, making only 27% of homes “easily accessible” by residents [37], although—under the 2006 UN Convention on the Rights of Persons with Disabilities—the Member States have the obligation to identify and remove all obstacles to accessibility, including housing [38].

Domestic accidents are also closely related to the structure and functional layout of the accommodation, as well as the personal characteristics of the inhabitants (e.g., age, habits, etc.). Considering the available evidence, the WHO [17] strongly recommends housing interventions aimed at preventing domestic accidents, for example, eliminating obstacles and adopting design solutions that take into account the sizing of the premises [3,15,39].

For the reasons described above, the reflection on living spaces, over the years, has expanded in the health sector [3,10,14,17,19,27,39], but also in the technical and economic sectors [6,7,8,15], due to the need to offer an adequate and sustainable housing supply.

Considering the issues described above, this paper, as part of a debate on housing’ dimensional requirements interesting different countries [6,7,15], analyses and compares housing condition indicators of some European countries and their housing dimensional standards to evaluate their ability to answer population needs in terms of health and affordability and to define updated indication for Italian regulation.

## 2. Materials and Methods 

The study presented in this paper was conducted as part of a research project funded by the Italian Ministry of Health, aimed to update health performance objectives for the construction and/or renovation of buildings in Italy [40]. In particular, the study focused on the analysis and comparison of Italian housing standards—defined by Health Ministerial Decree dated 5 July 1975—with those of some European countries [41] to investigate the aspects related to the housing dimensional standards and how these have been dealt with, within the regulations. The interest of the study is to identify performance objectives capable of ensuring a healthy and sustainable home that considers the new needs of the occupants in terms of health, equity and affordability.

**Figure 1:**
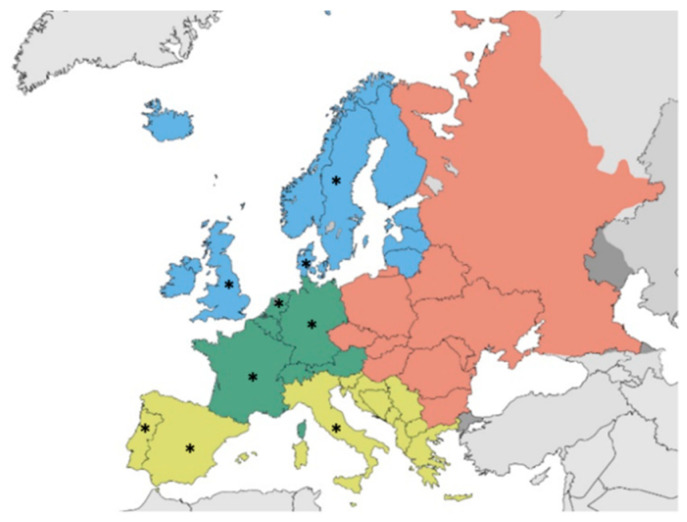
Map of North, Central and South Western European countries. * countries selected.

The study carried out in 2018 and updated between May and August 2020, regarded the research and review of Building codes (BCs) of a sample of West-European countries, following the methodology already applied in previous studies, although with different aims [42,43]. To obtain an updated indicative picture of dimensional requirements in North, Central and South of Western European countries, nine countries were selected, among those most populated (Figure 1) and similar from a socio-economic and development point of view.

Table 1 reports the selected countries, their population and density. Over 365 million people live in these countries, accounting for about 89% of the West European population. They overall represent about half (47.6%) of the resident population of the whole European continent in 2019 [44].

The BCs were found in the governmental websites of the different member states, as well as in “Energy Efficiency in Buildings” website (http://www.buildup.eu, accessed on 15 May 2020).

The BCs’ analysis, focused on hygienic and sanitary housing requirements in terms dimensional standards and functional characteristics of dwellings, compared the following aspects, if reported:

- minimum housing size;
- minimum living space for inhabitant;
- minimum room size for inhabitant;
- rooms’ minimum height.

To obtain a more reliable evaluation, the BCs were selected and revised from two researchers independently. All the investigated data were reported in data sheets by each researcher and later compared and discussed.

In the case of countries with federal structures, their analysis focuses on a single province or region, selected following two major criteria: regulation update and availability of detailed quantitative parameters to compare with the other selected countries. This is the case with Germany and Spain, where the analyses focused on Hessen and Catalonia, respectively.

About the United Kingdom, data refer to England and Wales.

At the same time, to better understand and justify the differences among the various building regulations, some basic housing indicators of each country were selected and compared.

The analysis focused on three main points:

1. housing characteristics, in terms of average housing size, building type (flat, detached, or semi-detached house), housing deprivation (dwellings with poor amenities), to picture the housing supply of each country.
2. distribution of housing tenure status and costs due to different tenure conditions (ownership or tenantship) by country. Housing costs refer to monthly expenditure that is connected to living in a property (e.g., mortgage, taxes, costs of utilities or rental payments for tenants, etc.), to understand housing affordability.

Data were selected from Eurostat database of the European Commission [45] regarding “statistics on housing conditions” and refers to the last data available.

## 3. Results

### 3.1. Regulatory Documents Used for Comparative Analysis

Table 2 shows the regulatory documents analyzed for the comparative analysis of the investigated countries; furthermore, Table 2 describes the prevalent approaches used to define each standard (functional or prescriptive or performance formulation).

Building codes in the Federal Republic of Germany fall under the responsibility of state governments [42,43,46] and are based on a central Model Building Ordinance model. The regulations of the State of Hessen were examined for this work: *Hessische Bauordnung* (HBO) 2012 [47]. The HBO requirements are mostly functional statements and only for some topics (e.g., minimum ceiling height, etc.) specific requirements and operational notes are introduced. In the BC, there is no indication of verification methods, but reference to specific and national rules [42,43,46] is implicit.

As far as England and Wales is concerned, the legislation referred to in this work is represented by the Building Act 1984, as amended by the Deregulation Act 2015 [48] by the Building Regulations 2010 Building (Amendment) Regulations 2016 [49]. The Building Act provides the legislative basis for the Building Regulation, which defines short functional requirements [42,43,46,50].

For France, the normative documents analysed are *Décret* n°2002-120 (Version consolidée 7 septembre 2020) [51], which indicates the essential characteristics that should be fulfilled in order to have proper housing, and a national technical reference standard, *Code de la Costruction et de l’Habitation* (Building and housing code) [52], which is mainly prescriptive.

In Italy, the minimum dimensional standards are indicated in the Ministerial Decree, 5 July 1975 [53] supplemented at municipal level by local building regulations, which often adopt parameters that differ considerably from the main regulations [54].

For Spain, the regulatory documents analysed are Royal Decree 314/2006—*Código Técnico de la Edificatión* (CTE) [55] and the Basic Documents annexed to it (mainly the Basic Document SU—*Segurided de utilizacion y accesibilidad* 2018 and Basic Document HS—*Salubridad* 2017) [56,57]. The Basic Documents contain procedures, technical rules and examples of solutions to determine whether the building meets the established performance levels. These standards are then supplemented at regional level with additional regulatory documents; for the present work, for example, *“Decreto 141/2012, de 30 de octubre, por el que se regulan las condiciones mínimas de habitabilidad de las viviendas y la cédula de Habitabilidad”* [58] of the Catalan Region was taken into consideration.

Regarding the Netherlands, the Building Decree [*Bouwbesluit*] 2012 [59] was selected, whose introduction in 2003 led to the national standardisation of all technical regulations. The BC contains the performance standards for buildings, and for each of them a functional statement is provided, describing the purpose of the performance requirement, and operational requirements including minimum values, which indicate the minimum level of performance to be achieved, and the methods to determine them [46].

The minimum dimensional standards in Portugal are contained in the General Regulation of Urban Construction (*Regulamento Geral das Edificações Urbanas)* (RGEU) 2009-01-01 [60] and subsequent additions and modifications (which generally include general provisions for building, health, safety and aesthetics) [61], supplemented, as in Italy, by technical and sector regulations. The RGEU adopts a prescriptive formulation of dimensional parameters.

For Sweden, the legislation used in the comparative analysis is as follows: Boverket´s Building Regulations 2016 and Boverket´s Building Regulations 2018 [62]. The Building Regulations implement the provisions of the Planning and Building Act (1987: 10) [42,43,46] and contain mainly functional statements and general recommendations. Most of the provisions are performance requirements (e.g., design for accessibility) and some operational requirements (e.g., ceiling height). General recommendations include technical examples, testing methods and some explanatory information [42,43,46].

As regards Denmark, the following reference legislation was selected: Executive order on building regulations 2018 (BR18) [63]. In the Danish BC there are mostly mandatory functional indications accompanied by references to specific standards and technical codes.

In conclusion, the regulatory documents of Denmark, Germany, the Netherlands, Spain, and Sweden define their standards mainly based on performance, with some prescriptive requirements. Those of France, Italy and Portugal identify prescriptive standards, accompanied by more recent technical performance standards, specific for each sector. Finally, UK regulation does not indicate parameters but provides some functional indicators.

### 3.2. Minimum Dimensional Standards

The analysis of regulatory documents shows different approaches to the definition of dimensional standards in the European countries considered. In most of the countries analysed in this study, the development of housing is undertaken within a framework of fixed minimum interior spaces [7]. The regulations and BCs indicate the amount of living space that must be provided, the minimum acceptable ceiling heights, and, in some cases, the room’s side length and the volumes. The various regulations and/or indications are handled in different ways and the criterion with which in which space is measured (if the surface of the interior walls, for example, is included in the floor space or in the total living space) varies from country to country. There are, however, some exceptions: some countries where—although there are several rules that affect the quality of construction—the surface area of houses and ceiling heights are not subject to any legal minimum written in national legislation (e.g., England and Wales, Denmark).

Table 3 shows the minimum dimensional standards available in each examined country. The notes add some specific aspects of dimensional requirements, described in each norm. The table and the notes show that these standards vary from one country to another considerably.

It must be argued that a direct comparison between the dimensional standards of the different countries is quite difficult to attain, both for the different setting of the standards and for the definitions of the individual parameters. For example, the living space, defined in a rather generic way in many BCs of the different European countries, in Italy is clearly explained in the Circular Letter of the Ministry of Public Works dated 23 July 1960, n.1820 as “total surface of useful rooms” [64].

The regulations of the England and Wales [48,49] and Denmark [63] do not identify minimum size standards either for the living space or for the different rooms, and, for this reason, are not included in Table 3.

As far as the minimum heights are concerned no specific standard is indicated in Denmark code, while the minimum limit has been abolished in the English national codes (the minimum limit of 2.14 m is set by local regulations).

The regulation of Germany (Hessen) [47] (Table 3) too does not identify minimum size standards for the different housing environments; instead, as regard the minimum height of the rooms, the standard is set at 2.40 m for the main rooms and 2.20 m for basements and garrets.

As shown in Table 3, in France, a minimum standard for living space measured in square metres per inhabitant (14 m^2^ per inhabitant) is indicated and includes the entire net surface area of the accommodation. The French regulation [51,52] does not identify minimum standards for the studio apartment but indicates its volume parameter (33 m^2^ per inhabitant). As far as the division of space inside the dwellings is concerned, excluding bathrooms, French regulations make an open-plan configuration possible as they provide neither indications on the separation of rooms nor specific standards for individual rooms, except for the single bedroom which must have a minimum surface area of 9 m^2^ and in any case not less than 7 m^2^. The minimum height in the accommodation is 2.20 m (Table 3).

In Italy, as in France, the dimensional standard of the living space is measured in m^2^ per inhabitant: the minimum requirement is 14 m^2^ each for the first 4 occupants (Table 3), which is reduced to 10 m^2^ for each additional occupant. While there is no minimum cubage as far as studio apartments are concerned, the MD of 5 July 1975 [53] provides dimensional indications for dwellings in proportion to the number of inhabitants (net surface area: 28 m^2^ per 1 occupant, 38 m^2^ per 2 occupants).

A closer examination of the regulations, in relation to the dimensional standards for dwellings, reveals that the Italian regulations are among the strictest and, at the same time, most protective for the health of the inhabitants, defining a minimum of 14 m^2^ for the living room (excluding the kitchen), 9 m^2^ for a single room (Table 3) and 14 m^2^ for a double room. As far as the division of space within dwellings in Italy is concerned, it is compulsory to provide separate spaces (except in the case of studio apartments) and the main rooms must be separated from the bathrooms by a hallway. As far as the minimum ceiling height is concerned, the standard is 2.70 m in the main rooms and 2.40 m in the service rooms (Table 3).

The Spanish regulations analysed [55,56,57,58] indicate different standards for existing and new buildings. The minimum dimensions prescribed in new buildings for living space are 20 m^2^ and include the living room and kitchen. In Spain there are no mandatory separate spaces and in fact there are no dimensional indications on the living space, except for the single bedroom whose size must be 6 m^2^ (Table 3), which is reduced to a useful surface of not less than 5 m^2^ in pre-existing buildings. As regards studio-apartments, the Decree 141/2012 in force in the Catalan Region (Spain) indicates a minimum area (net surface) of 20 m^2^. The minimum standard for ceiling height is 2.50 m in the main rooms and 2.20 m in service rooms (Table 3).

The Netherlands legislation [59] sets a minimum size standard for the living space of 18 m^2^ for new buildings. The legislation also prescribes that at least 55% of the space inside homes must consist of living space (living room, kitchen, bedrooms); this requirement was introduced to limit service spaces (bathrooms, corridors, circulation spaces, technical spaces).

As far as the division of space inside homes is concerned, The Netherlands has a standard for the living room (e.g., living room, bedroom) of 11 m^2^ for new buildings (Table 3), which is reduced to 7.5 m^2^ for the existing buildings. The Netherlands BC indicates a further parameter, i.e., the minimum width of living rooms, which must be ≥3 m for new buildings and ≥2.40 m for existing buildings. This approach is very interesting, also with respect to the definition of a minimum surface area, because its main objective is to create usable, furnished, and functional space. The Building Decree also indicates different minimum dimensions for toilets (0.90 × 1.20 m) and bathrooms (1.6 m^2^ with a width of at least 0.8 m); if combined, the minimum area of the room must be 2.20 m^2^ with a side of at least 0.90 m. Finally, the minimum standard for ceiling height is 2.60 m in the main rooms and 2.20 m in the service rooms.

The RGEU [60] of Portugal does not provide information on the living space, whereas it provides separate minimum standards for main rooms and the bathroom. In Portugal, the living room must be at least 10 m^2^ (plus 6 m^2^ for the kitchen if necessary), the first bedroom at least 10.5 m^2^ and can accommodate up to two people (9 m^2^ for the second double bedroom and 6 m^2^ for the single bedroom) and the bathroom at least 3.5 m^2^. As far as studio-apartments are concerned, Portuguese legislation identifies minimum dimensions for one person of 35 m^2^, considering the gross floor area. And finally, the minimum standard for ceiling height is 2.70 m in the main rooms (as in Italy) and 2.20 m in the service rooms (Table 3).

The Swedish BC [62] identifies performance requirements relating to the dimensions of dwellings, specifying functional indications, minimum furnishings, etc., but not specifying numerically the minimum dimensions of the habitable rooms (Table 3). As regards the minimum ceiling height, the standard is 2.40 m (without distinction between main and service rooms) and 2.30 m for basements and garrets.

In the various countries, the regulatory documents subject to the comparative analysis impose the number of bathrooms (at least one bathroom) and the sanitary facilities they must have, except for the England and Wales.

### 3.3. Housing Characteristics Comparison in the Examined Countries 

As observed previously, some social and demographic changes are influencing housing demand: evolving family structures and a growing proportion of elderly people is resulting in an increasing number of homes being required to house a quite similar number of inhabitants. At local level, given the considerable investment that is required, it is often difficult to make rapid changes to the type and the number of houses that are made available to those looking for a new home [4,65]. In the examined countries existing building heritage is generally old, with a large number of constructions built before 1980 (in average, more than 65%) [65]. This means that several structural problems (e.g., inadequate fixtures causing waste of energy, indoor physical obstacles like stairs, space distributions variation) require relevant investments to be adapted to new need in terms of health and sustainability.

Housing average size of the real estate stock is generally acceptable (Table 4), as demographic changes have led to smaller household sizes, while most people, at least in low-income families [27], aspire to have more space to live in. As Table 4 shows, in average, house sizes in Denmark (118.1 m^2^), the Netherlands (106.7 m^2^) and Portugal (106.4 m^2^) result above 100 m^2^, while they are relatively smaller (and in any case with a average greater than 90 m^2^) in Sweden (99.7 m^2^), Spain (99.1 m^2^), Germany (94.3 m^2^), France (93.7 m^2^) and Italy (93.6 m^2^). Regarding the UK, in 2009, Williams reported an average dwelling size of 87 m^2^ [11]. This size of dwellings is likely to reflect, at least to some degree, population density and housing concentration in urbanized areas, but may also reflect variations in the price of land and housing, income distribution, as well as the housing stock available for rent or for purchase. For example, in Denmark (53.2%), Sweden (44.4%) and France (42.4%), a relatively high share of the population lives in detached homes, while in Spain (64.9%), Germany (53.6%) and Italy (52.6%) it is more common for people to live in flats. In the UK, semi-detached homes are commonest (60.8%) (Table 4).

The housing deprivation index measures the percentage of the population living in dwellings with poor amenities (e.g., leaking roof; no bath/shower, or no indoor toilet; dwellings that are considered too dark, etc.). Housing deprivation, in 2018, ranges from 5.0% in Italy to 1.3% in the Netherlands. One of the key dimensions in assessing the housing quality is the availability of sufficient space in the home also. The indicator used to evaluate the space availability is the overcrowding rate, as defined by Eurostat, that calculates the percentage of people living in an overcrowded household. A household that does not have at its disposal a minimum number of rooms is considered overcrowded, depending on the household composition [66]:

- one room for the household;
- one room per couple in the household;
- one room for each single person aged 18 or more;
- one room per pair of single people of the same gender between 12 and 17 years of age;
- one room for each single person between 12 and 17 years of age and not included in the previous category;
- one room per pair of children under 12 years of age.

Therefore, for Eurostat, room size does not matter. In its view, the main factor affecting overcrowding is the number of rooms. In 2018, the highest overcrowding rate among the analyzed countries in this study (Table 4) is recorded in Italy (28.3%), while the United Kingdom (4.8%), and The Netherlands (4.8%) recorded the lowest ones.

Housing markets show significant differences in tenure status, i.e., in the percentage of people owning their homes. Table 5 provides an overview of the ownership status of owner-occupied dwellings in 2018 for the investigated countries.

The highest rates of home ownership are recorded in Southern European countries such as Spain (76.2%), Italy (72.4%) and Portugal (73.9%). On the contrary, the lowest percentage of owned properties is registered in Germany (51.1%). As Table 5 shows, all the other countries show home ownership rates ranging between 60% and 70% (Denmark 60.8%, Sweden 63.6%, France 64.1%, The Netherlands 68.9%, UK 65.2%).

At the same time, in the United Kingdom (37.7%) and Spain (38.1%) more than one third of the population, living as tenants with market price rents, spend more than 40% of their disposable income on housing, followed by Spain (38.1%), Italy (29.1%) and Denmark (28.9%), while France (14.9%) and Sweden (18.9%) show the lowest percentage of overburden [67].

## 4. Discussion

The comparative analysis carried out allowed us to identify convergences and divergences in the Building Codes and standards taken into exam. There is a diversified approach among European countries: from a market-oriented approach (e.g., England and Wales), where minimum dimensions are not defined, and a prescriptive approach (e.g., Italy), to a functionality-oriented approach (e.g., the Netherlands).

As introduced before, attitudes towards minimum standards of space are specific to places and experiences: they come to reflect fundamental beliefs about the usefulness and purpose of housing. Where housing is seen as an investment good, standards are likely to be seen as a threat; where investment is a weaker driver than the market and the quality of houses is judged on their long-term utility and adaptability, rather restrictive standards are set (e.g., in Italy) [7]. With respect to the above, the abolition of minimum standards in the England and Wales and the weight given to the functionality of space are strategies dictated by market trends. The market determines what is built: or, more specifically, standards are determined by what people are willing to buy [7]. For example, in the UK, for 15.1% of the total population the housing costs exceed 40% of their equivalized disposable income; it is the highest percentage among examined countries and, probably, it can contribute to explaining why the average size of dwelling is lower than in other states.

Most of the regulations currently in force focus, however, on more established housing models, without considering changes in lifestyle or requirements (singles, childless couples, students or seasonal workers, the elderly alone) [50,68]. The comparative analysis clearly shows that the dimensional standards are very different from each other in terms of both form and rigour and completeness.

The Italian legislation dictates stricter requirements than those of other countries, but, at the same time, protects the more vulnerable classes from the point of view of living spaces, defining a larger minimum standard in terms of housing size. These regulations, however, do not always guarantee an adequate level of quality in the home. In fact, 5% of Italian population lives in deprived housing (e.g., dwellings with a leaking roof, or no bath/shower, or no indoor toilet, or dwellings that are considered too dark) and this percentage is the highest among all investigated countries (see Table 4).

Furthermore, living spaces themselves, often, do not fully satisfy the needs of users and should be adapted to meet new functional requirements, also for the psychological and physical well-being of the inhabitants. For example, in Japan, the average floor area per person passed from 17.3 m^2^ in 1963 to 30.9 m^2^ in 1993, which is an impressive improvement [6].

In a 2005 study [69], the authors elaborate a comparison of housing size for EU15 by analysing average sizes of new housing, number of rooms and average size of individual rooms. The comparison showed that the average floor area of new homes in the UK, equal to 76 m^2^, was substantially smaller than in any other country. In fact, the average size of new houses in Denmark (137 m^2^) was almost twice as large as in the UK. In Belgium, Greece, and the Netherlands the average was 50% larger (119 m^2^, 126.4 m^2^, 115.5 m^2^ respectively) and almost 50% larger in Germany (109.2 m^2^) and France (112.8 m^2^).

The average floor area of new homes in Italy (81.5 m^2^) was the second lowest among EU15 countries, but it can be explained in part by the high costs of houses, especially in metropolitan areas, as well as by the progressively reduced number of household components, due to the demographic drop.

Regarding the UK, Evans [69] reports that compared to other countries, new houses built have a relatively high number of rooms (4.8); the consequence is that the average size of the rooms in new homes, just over 15 m^2^, is smaller than in the other countries. In comparison, new houses in Italy have a limited number of rooms (3.8) with respect to the floor area of the house and the average room size is larger (21.4 m^2^). The same is for Denmark with a limited number of rooms (3.5) and an average room size of 39.1 m^2^. Portugal and France also impose detailed regulations, although less cautious for the inhabitants than Italy, while in the Netherlands, we find a balanced system between clear performance objectives, health protection and design freedom [50].

The complete abolition of minimum dimensional standards, for example in England and Wales, has caused considerable controversy. Obviously, freedom from prescriptive minimum size requirements allows the market to meet the needs of a more diverse range of families in the present, but very often, the houses themselves are not adequate for the future because they are unable to adapt to the changing needs of families throughout life. Furthermore, the lack of legal minimum space requirements has led to a reduction in room size; this could contribute to determining serious consequences for the physical and psychological health of the inhabitants. As argued by Williams [11], for the purposes of studying space per person, information about dwelling sizes, measured by floor area, is very useful. It is a measure of how much ‘private’ indoor space people consume. Houses with the same number of rooms can vary considerably in floor space.

At the same time, some authors [7] criticize the use of floor space averages as a housing quality measure for several reasons. Firstly, the arrangement of indoor space is potentially more significant as it improves its utility. Secondly, statements about space need to be linked to occupancy levels: relatively large houses can have limited utility if they are overcrowded [7].

Linked precisely to the size and design of the accommodation is the problem of overcrowding. This not only depends on the number and size of rooms and the number of people sharing the dwelling, but also on their age, relationship, and gender [3,18]. 

As reported in Table 4, in 2019, Italy showed the highest overcrowding rate among the EU states analyzed, equal to 28.3%. This comparison can be confusing because it does not consider that in Italy the minimum room size is defined by the regulations. As shown in Table 3, in Italy, the standard defines a minimum room size, based on use, that is much higher than those foreseen in other countries (e.g., a double room of 14 m^2^ and a living room of 14 m^2^). Therefore, considering that Eurostat bases the evaluation of overcrowding on the number of rooms available in the accommodation and not on their size, with the same size, in Italy, the number of rooms available will always be lower than in other countries [10,66].

In Italy, the National Institute of Statistics (ISTAT) measures the crowding of dwellings in terms of the number of family members per square meter of the dwelling (expressed in values per 100 m^2^) [70]. We speak of overcrowding if there is a greater number of people present in the home than that resulting from the application of the parameters indicated in Articles 2 and 3 of the Health Ministerial Decree of 05.07.1975 [53]. These articles refer to dimensional criteria of the rooms, an element that, as mentioned above, does not emerge in the definition of overcrowding by Eurostat and many other international standards, which focus on the number of rooms rather than their size.

According to the 15th census of Italian population (2013), on average, in Italian homes the residents have 40.7 m^2^ per occupant [71], a value similar or higher than that observed in other countries [6,11].

It is now widely recognized that housing (both the product itself and the production process) needs significant improvements [7,8,15]. Space within housing, living space and the impact it can have on the health of inhabitants is, to date, a key point in the debate on housing. From the technical–architectural point of view, therefore, it is necessary to rethink living spaces by focusing attention on some fundamental characteristics that make for adequate accommodation: flexibility, adaptability and furnishing of the premises and full usability and accessibility of the spaces [72,73]. The small size of the housing, combined with poor construction quality and general standardisation of housing products, is very often a limiting factor for flexibility and adaptability [74].

As mentioned previously, in recent years, awareness of the importance of housing quality for physical and mental wellbeing has grown considerably [17] and the recent Covid-19 pandemic has highlighted the absolute need to rethink living spaces by focusing on certain elements necessary to protect the indoor wellbeing [3,18]. In fact, even in the absence of other pollutants, indoor air quality becomes progressively worse the more people there are in a room, especially if the availability of space (volume) per person is scarce. Excluding the already-described risk of easier spread of an infectious disease, stuffy air (mostly due to high concentrations of carbon dioxide) can cause headaches, difficulty in concentration and drowsiness, thus impairing learning performance, without considering the psychological implication of the lack of privacy and individual space [19,27,75]. These problems are amplified by the application of sealed fixtures to save energy. Of course, some technical solutions (e.g., using air change systems) may solve the air quality problems, but they are not able to solve the quality of living and privacy problems. The availability of adequate space, its adaptability and flexibility [3,18,76], are of fundamental importance in terms of their impact on the health and safety of inhabitants and further research is necessary to find satisfactory solutions for the needs of the various population subgroups. It is also necessary to quantify the percentage of housing stock not satisfying the standards and the number of people living there, considering the variability between countries.

In a recent paper [77], it was proposed to prioritize a dimensioning of the rooms in the home in relation to the number of occupants (m^2^ per inhabitant) and their use, rather than giving importance to a clear separation of rooms (as in the Italian legislation) or the number of rooms in the home (e.g., Portuguese legislation).

Considering the recent trends, many standards should be reviewed, and the relevant legislation integrated, updated, and simplified [78,79,80]. Italian local building regulations, for example, are obsolete and heterogeneous and the subsequent derogations introduced at national, regional, and local levels represent a serious obstacle to the promotion and conservation of public health [22,81]. In any case, we agree with Morgan and Cruickshank [30] who observed that “*converting an understanding of how different people use their homes into a single number will never fully reflect the complex needs of real people*”.

## 5. Conclusions

In conclusion, the appropriate amount of internal space definition for a dwelling is a complex issue. Several factors related to culture, economics, society, affordability, building heritage, influence housing supply and demand, other than health issues. Therefore, we think this topic needs to be assessed with a multidisciplinary and transdisciplinary approach in both research and practice, because of the complexity and wideness of its components.

The international trend anticipates the increase of the average housing size, at least in developed countries, both for objective (e.g., sufficient circulation space) and subjective reasons (e.g., claustrophobia problems, infectious diseases prevention, but also private space for working). Today, it is well known that people’s perception of internal space is only partially correlated to its amount, being also related to age and gender.

Now, there is limited research on the appropriate size of housing and on its relevance in terms of health, safety, and people’s wellbeing. The needs evolve during time and housing regulations are frequently rigid and unable to be adapted; lower income population groups are those who mainly pay the consequences for this.

At the same time, it must be argued that the aspects relating to the size of housing, combined with the adequate sunlight and external view, are of particular importance today, also considering the criticalities overwhelmingly emerged during the recent Covid-19 pandemic. Regarding the Italian regulation, the comparative analysis carried out allowed us to identify the strengths and weaknesses of the MD 5 July 1975, which we highlighted in the previous section, and possible indications for its updating, in line with the provisions of the BCs of the countries studied. The study shows, in fact, the need to integrate some key concepts of the MD 5 July 1975, such as, for example, the one relating to living spaces. It is necessary to introduce a general concept of living spaces to the Italian MD, defining its performance objectives (e.g., room sizing, air volume, etc.) and its minimum performance (usability, accessibility and furnishing), eliminating rigid divisions by rooms.

In this view, the MD 5 July 1975 should be updated by defining only the overall living space in proportion to the number of inhabitants. In such spaces furnishing should be guaranteed in such a way as to favor internal accessibility, at least in terms of adaptability according to current regulations.

In conclusion, building healthy and safe housing is a complex issue and a multi-sectorial responsibility, achievable only if a contribution is made by all relevant players, since it needs of policy vision, health data, resources, and technical competences.

In Italy, the Covid-19 pandemic has placed the country’s profound housing crisis in the spotlight and the large variability between the regions and highlighted the need to address it in a systematic way, considering this important issue in the Country recovery agenda. At the same time, it will be also important and necessary to find innovative housing solutions in terms of spaces availability and their usability to respond to future emergencies.

## Figures and Tables

**Table 1:** Selected countries, population and population density. Source: https://www.populationpyramid.net/it/italia/2019 (accessed on 9 September 2020).

Countries	Population (Updated to 2019)	Density per/km^2^ (Updated to 2019)
Germany	83,517,046	233.69
United Kingdom	67,530,161	277.21
France	65,129,730	118.61
Italy	60,550,092	200.94
Spain	46,736,782	92.38
The Netherlands	17,097,123	411.58
Portugal	10,226,178	110.88
Sweden	10,036,391	22.43
Denmark	5,771,876	134.47

**Table 2:** Documents and regulations and their main characteristics.

Countries	Regulations and Documents	Main Characteristics: Approach to the standard
Germany	- Hessische Bauordnung (HBO) 2012 - Model Building Code	Mostly performance formulation, with some functional descriptions and specific requirements and prescriptive notes
United Kingdom (England and Wales)	- The Building Regulations 2010 Building (Amendment) Regulations 2016 - Building Act 1984, as amended by the Deregulation Act 2015	Functional formulation
France	- Décret n°2002-120 (Version consolidée au 7 septembre 2020) - Code de la Costruction et de l’Habitation (Building and Housing code)	Mainly prescriptive formulation
Italy	- Ministerial Decree, 5 July 1975 (and M.I. 1896 for the relevant parts still in force)	Prescriptive formulation
Spain	- Royal Decree 314/2006 - Código Técnico de la Edificatión (CTE) - Documento Basico SU—Segurided de utilizacion y accesibilidad 2018 - DECRETO 141/2012, de 30 de octubre, por el que se regulan las condiciones mínimas de habitabilidad de las viviendas y la cédula de Habitabilidad. Cataluña	Performance formulation, with some prescriptive parameters
The Netherlands	- Building Decree [Bouwbesluit] 2012	Mainly performance formulation
Portugal	- Regulamento Geral das Edificações Urbanas (RGEU) 2009-01-01	Prescriptive formulation
Sweden	- Boverket’s building regulations 2016 - Boverket´s building regulations 2018	Mainly performance formulation, with some functional and operational requirements
Denmark	- Executive order on building regulations 2018 (BR18)	Performance formulation, with functional descriptions and references to specific standards and technical codes

**Table 3:** Dimensional parameters of housing and living space in housing in the examined standards.

Countries	Living Space (m^2^)	Habitable Volume (m^3^)	Studio Apartment Area (m^2^)	Living Room (m^2^)	Bathroom (m^2^ per inh.)	Single Bedroom (m^2^)	Room Side Length (m)	Minimum Height (m)
Germany	-	-	-	−(1) Net floor area, living rooms: 10 m^2^. For houses with several bedrooms and living rooms, one room can be 6 m^2^.	-	−(1) Net floor area, living rooms: 10 m^2^. For houses with several bedrooms and living rooms, one room can be 6 m^2^.	-	2.40 Germany minimum height for attic and basement 2.20 m.
France	14 The minimum living area/inh is: 14 m^2^ for the first 4 people, 10 m^2^ from the fifth on for each additional one. The minimum volume/inh is: 33 m^2^ for the first 4 occupants, 23 m^2^ from the fifth on for each additional one.	33 The minimum living area/inh is: 14 m^2^ for the first 4 people, 10 m^2^ from the fifth on for each additional one. The minimum volume/inh is: 33 m^2^ for the first 4 occupants, 23 m^2^ from the fifth on for each additional one.	-	-	-	9 The minimum surface area is 9 m^2^ and, in any case, not less than 7 m^2^.	-	2.20
Italy	14 The minimum living area/inh. is 14 m^2^ for the first 4 inh.; 10 m^2^ from the fifth on for each more inhabitant.	-	28 In case of a studio apartment the minimum area is 28 m^2^ for a single occupant and 38 m^2^ for 2 occupants	14	-	9 The minimum single bedroom area is 9 m^2^, 14 m^2^ for the double room	-	2.70 Minimum height 2.40 m for service rooms
Spain	20 The minimum living space/inh is 20 m^2^, including common areas (living room, living room and kitchen).	-	20 The minimum usable floor area of dwellings is 20 m^2^ for buildings existing prior to the Decree 141/2012 (Cataluña)	-	-	6 In dwellings ≥3 rooms, one of them must be inscribed in a square of 2.60 m of side. In the other rooms and in dwellings ≤2 rooms, these can be inscribed in a square of 2.00 m on the side. The single bedroom must have a minimum area of 6 m^2^ for new buildings, 5 m^2^ for existing buildings. The double bedroom must have a minimum surface area of 8 m^2^, the triple of 12 m^2^.	-	2.50 Minimum height for service rooms 2.20 m
The Netherlands	18 (2) The minimum floor area/inh is 18 m^2^ for new buildings, specifying that the living area used as a sleeping and living area is > 55% of the total surface area of the accommodation (including accessory areas), 10 m^2^ in the case of existing buildings.	-	-	11 The minimum area is 11 m^2^ for new buildings, 7.5 m^2^ for existing buildings.	2.2 For new buildings (NB) the toilet must have an accessible space with an area of at least 1.65 m × 2.2 m; the fully accessible toilet must have an area of 1.6 m × 1.8 m. For existing buildings (EE) the Toilet must have a minimum surface area of 0.9 m × 1.2 m, with a minimum height of 2 m. A bathroom combined with toilets must have an area of at least 2.2 m^2^ and a width of 0.9 m. A fully accessible integrated bathroom must be at least 2.2 m × 2.2 m.	-	3 The minimum size of the room side must be 3 m for new buildings and 2.4 m for existing buildings.	2.60 The minimum height for service rooms 2.20 m
Portugal	-	-	35 The gross floor area is referred to as the gross floor area for single occupancy accommodation.	10 In addition to the 10 m^2^ of living room there are 6 m^2^ of kitchen.	3.5	10.5 (3) Each flat must have at least one bedroom of 10.5 m^2^ for 2 people, the second bedroom may have a surface area of 9 m^2^ for two people, in case of an additional room this may be a single bedroom of 6 m^2^.	2.1	2.70 Minimum height for service rooms 2.20 m
Sweden	- Dwellings with a residential area (> 55 m^2^; 35–55 m^2^; <35 m^2^) must be designed according to the number of people for which they are intended.	-	-	- Dwellings must be designed according to the number of people for whom they are intended.	-	- Dwellings must be designed according to the number of people for whom they are intended.	-	2.40 Minimum height for attic and basement 2.30 m.

Inh = inhabitants. (1) With reference to Hesse 1993. (2) The building code in the Netherlands is the only one that also shows minimum linear surfaces in terms of room width and depth. (3) In Portugal accommodation is divided according to the number of rooms available: T0 is a studio flat, T1 is a 1-bedroom flat, T2 with 2 rooms, etc. This logic is also present in the building code, divided by type of accommodation.

**Table 4:** Dwellings by type, average size and overcrowding (Eurostat, 2019).

Countries	Average Dwelling Size (m^2^)	Dwelling Type (%)			Severe Housing Deprivation Rate (%)	Overcrowding (%)
		Flat	Detached	Semi-Detached		
Germany	94.3	56.3	26.4	15.8	2.3	7.8
United Kingdom	87.0 *	14.8	24.0	60.8	1.9	4.8
France	93.7	33.9	42.4	23.5	2.7	7.7
Italy	93.6	52.6	22.8	24.4	5.0	28.3
Spain	99.1	64.9	12.9	22.0	1.5	5.9
The Netherlands	106.7	20.2	17.4	58.0	1.3	4.8
Portugal	106.4	45.7	36.9	17.3	4.1	9.5
Sweden	99.7	46.2	44.4	9.2	2.9	15.6
Denmark	118.1	33.2	53.2	13.4	3.2	10.0

* this value is updated to 2009.

**Table 5:** Housing ownership, housing cost overburden rate, analyzed by tenure status and.

Countries	Home Ownership (%)	Housing Cost Overburden Rate				
		Overburden Rate (%)—Total Population	Owner-Occupied with Mortgage or Loan	Owner-Occupied without Mortgage or Loan	Tenant, Rent at Market Price	Tenant, Rent at Reduced Price or Free
Germany **	51.1	14.2	8.6	8.6	20.9	16.1
United Kingdom *	65.2	15.1	5.1	7.0	37.7	20.3
France **	64.1	4.7	0.7	0.6	14.9	8.9
Italy **	72.4	8.2	3.3	2.6	29.1	8.3
Spain **	76.2	8.9	3.5	2.6	38.1	10.1
The Netherlands **	68.9	9.4	2.2	4.0	25.6	7.8
Portugal **	73.9	5.7	3.0	2.0	25.8	4.9
Sweden **	63.6	8.3	1.7	6.4	18.8	0.0
Denmark **	60.8	14.7	5.2	7.1	28.9	-

* Values updated to 2018. ** Values updated to 2019.

## Data Availability

Data were selected from Eurostat database of the European Commission [45] regarding “statistics on housing conditions” and refers to the last data available. https://ec.europa.eu/eurostat/data/database (accessed on 16 December 2020).
